# Peroxo Species Formed in the Bulk of Silicate Cathodes

**DOI:** 10.1002/anie.202100730

**Published:** 2021-03-24

**Authors:** Zhenlian Chen, Bjoern Schwarz, Xianhui Zhang, Wenqiang Du, Lirong Zheng, Ailing Tian, Ying Zhang, Zhiyong Zhang, Xiao Cheng Zeng, Zhifeng Zhang, Liyuan Huai, Jinlei Wu, Helmut Ehrenberg, Deyu Wang, Jun Li

**Affiliations:** ^1^ Key Laboratory of Optoelectronic Chemical Materials and Devices School of Chemical and Environmental Engineering Jianghan University Wuhan China; ^2^ Ningbo Institute of Material Technology and Engineering Chinese Academy of Sciences Ningbo China; ^3^ Department of Chemistry University of Nebraska–Lincoln Lincoln NE USA; ^4^ Institute for Applied Materials (IAM) Karlsruhe Institute of Technology (KIT) Hermann-von-Helmholtz-Platz 1 Eggenstein-Leopoldshafen Germany; ^5^ Institute of High Energy Physics Chinese Academy of Sciences Beijing China; ^6^ Stanford Research Computing Center Stanford University 255 Panama Street Stanford CA USA

**Keywords:** ab initio calculations, lithium-ion batteries, oxygen redox, peroxo formation, X-ray spectroscopy

## Abstract

Oxygen redox in Li‐rich oxides may boost the energy density of lithium‐ion batteries by incorporating oxygen chemistry in solid cathodes. However, oxygen redox in the bulk usually entangles with voltage hysteresis and oxygen release, resulting in a prolonged controversy in literature on oxygen transformation. Here, we report spectroscopic evidence of peroxo species formed and confined in silicate cathodes amid oxygen redox at high voltage, accompanied by Co^2+^/Co^3+^ redox dominant at low voltage. First‐principles calculations reveal that localized electrons on dangling oxygen drive the O‐O dimerization. The covalence between the binding cation and the O‐O dimer determines the degree of electron transfer in oxygen transformation. Dimerization induces irreversible structural distortion and slow kinetics. But peroxo formation can minimize the voltage drop and volume expansion in cumulative cationic and anionic redox. These findings offer insights into oxygen redox in the bulk for the rational design of high‐energy‐density cathodes.

## Graphical abstract

Peroxo moieties formed in the bulk of carbon‐coated Mn‐doped Li_2_CoSiO_4_ are characterized by concurrent signatures in the form of an O‐K pre‐edge peak (XAS) and a peroxo O–O stretching mode (Raman). Peroxo formation proves to be a reversible oxygen redox mechanism for the development of high‐energy‐density cathodes in real‐world battery applications.

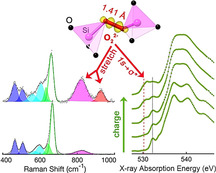

## Introduction

Oxygen redox (or anionic redox) in Li‐rich oxide cathodes offers a new way to increase the energy density of lithium‐ion batteries for applications in battery‐driven electric vehicles and green power grid stations.^[1]^ Unlike in oxygen evolution reaction (OER) and oxygen reduction reaction (ORR) on surface, oxygen ions are tightly bonded in the oxide lattice. Oxidation of the oxides indeed results in oxygen release and even structural collapse at elevated voltages.^[2]^ The form of the oxidized oxygen species is still debated heavily between two prevailing structural models, electron‐hole/oxygen‐hole^[3]^ or (O‐O)^*n*−^ (*n*=1–2) dimer species/O_2_ molecule.^[4]^ A new criterion is proposed to strictly distinguish anionic redox from cationic redox.^[5]^ Accordingly, the previously suggested 2.5 Å peroxo‐like O_2_^*n*−^ dimer is excluded as a valid oxidized product of oxygen redox reaction.^[5]^ A variety of O‐O dimers including detached O_2_/Li_2_O_2_ have been proposed for delithiated Li_2−*x*_MnO_3_ by first‐principles calculations.^[6]^ Two recent experiments suggest that molecular O_2_ trapped in the bulk is responsible for the voltage hysteresis in Li_1.2_Ni_0.3_Mn_0.54_O_2_.^[7]^ So far, very few cathode candidates have presented reversible oxygen redox at 4 V versus Li/Li^+^.[^1b^, ^8^] Structural disorder accompanied with oxygen oxidation has been suggested to be the common origin of voltage decay and slow kinetics.^[9]^ However, even the advanced characterization technique, resonant inelastic X‐ray scattering (RIXS), is still insufficient in determining whether the terminal‐oxo ligand or O‐O dimer dominates oxygen redox in Li/Na‐rich oxides.[^5^, ^9a^, ^10^] While antisite‐cation‐vacancy formation (ACVF) has been suggested as a key pathway to activate oxygen oxidation,^[5]^ it is still unclear why lattice oxygen transforms to a variety of oxidized species and what determines the redox pathway.

We notice that the oxygen redox activity in Li_2_Ir_1−*y*_Sn_*y*_O_3_ and Li_2_Ru_1−*y*_Sn_*y*_O_3_[^1a^, ^5^] shows dependence on non‐active element Sn. At stoichiometry *y*=0.5, Li_2_Ir_0.5_Sn_0.5_O_3_ delivers the highest capacity from oxygen species. This work turns to silicate Li_2_CoSiO_4_ that consists of light elements from the same groups (Ir, Sn) at equal ratio to gain insights into the oxygen transformation and electron transfer in oxygen redox. Previous works find cationic redox cannot support even one Li^+^ extraction in Li_2_CoSiO_4_.^[11]^ Recently, doped Li_2_CoSiO_4_ samples deliver over 300 mAh g^−1^ charge capacity and 220 mAh g^−1^ discharge capacity around 4 V in the first cycle,^[12]^ similar to Li‐rich oxides in delivering extra lithium capacity beyond one Li^+^ extraction.

This work utilizes synchrotron X‐ray absorption spectroscopy (XAS) and Raman spectroscopy to demonstrate the concurrently electronic and vibrational spectroscopic evidence of peroxo formation amid the second Li^+^ extraction at 4 V versus Li/Li^+^ in carbon‐coated Mn‐doped Li_2_CoSiO_4_. First‐principles calculations reveal that electron localization is the driving force for the oxygen dimerization in the bulk. The covalence between the binding cation and the O‐O dimer determines the oxygen evolution pathway. The agreement between experimental and first‐principles simulated X‐ray absorption near edge structure (XANES) suggests a criterion to ascertain which oxidized oxygen species dominates the oxygen redox reaction. With clarification of oxidized species, a new paradigm can be established for rational designs of cumulative cationic and anionic redox to achieve reversible high energy‐density by incurring peroxo formation.

## Results and Discussion

**Figure 1:**
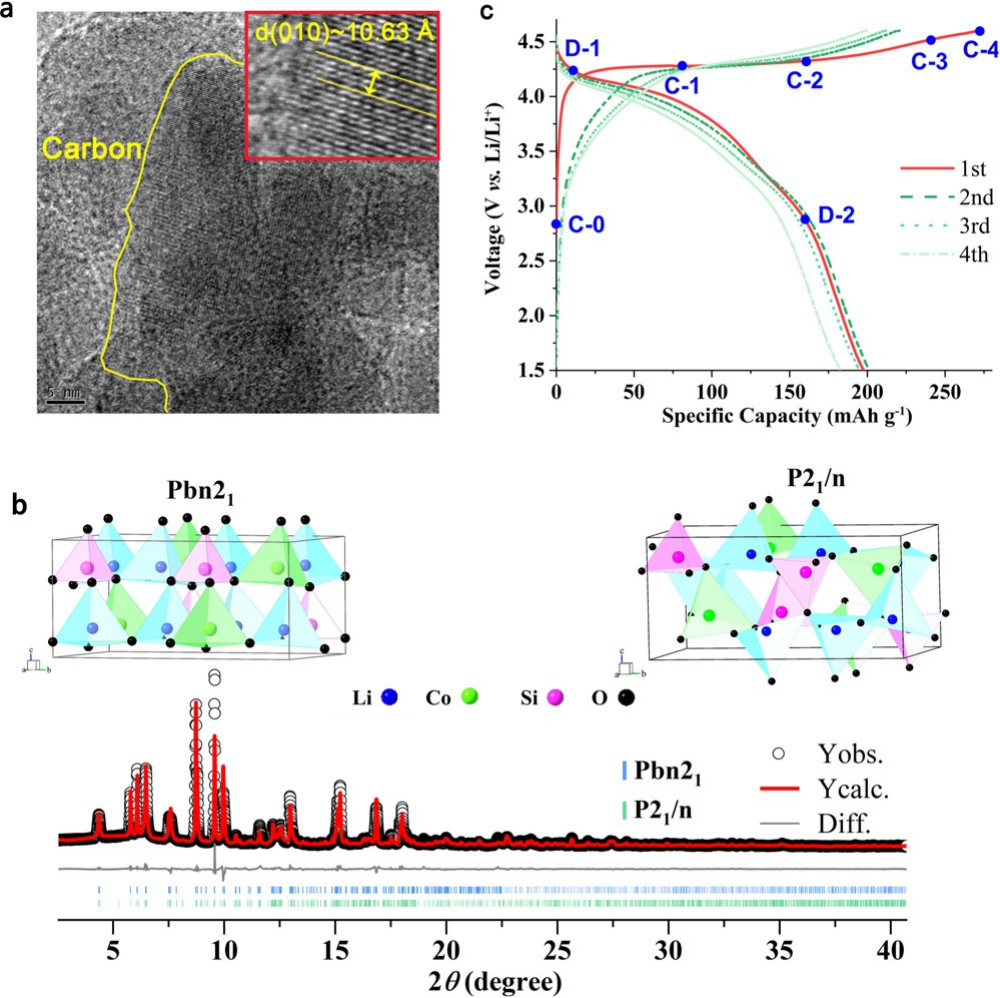
Morphology, crystal structure, and electrochemical performance of Mn‐LCSO. a) HR‐TEM image. b) Rietveld refinement against synchrotron XRD pattern (*λ*=0.41311 Å) of an as‐synthesized sample using polymorphs *Pbn*2_1_ and *P*2_1_/*n*. c) First four charge–discharge curves for Mn‐LCSO between 1.5–4.6 V vs. Li/Li^+^. Delithiated samples: C‐0 to C‐4 for charge capacity of 0, 80, 160, 240 and end of charge (mAh g^−1^), D‐1 and D‐2 for discharge capacity of 10 and 160 (mAh g^−1^), respectively.

Figure 1 a shows a high‐resolution transmission electron microscope (HR‐TEM) image of the as‐synthesized carbon‐coated Li_2_CoSiO_4_ with 6.25 % Co substituted by Mn (labelled as Mn‐LCSO). Mn is introduced to improve the electrochemical performance as do P‐, Al‐ and V‐doping shown in our recent works.^[12]^ The inductively coupled plasma emission spectroscopy confirms that the actual composition agrees well with the target composition of Li_2_Co_0.9375_Mn_0.0625_SiO_4_ with uniform Mn distribution, c.f., Figure S1 (in SI). The Rietveld refinement against synchrotron XRD pattern shown in Figure 1 b and Supplementary Table I suggests the dominant polymorph is β_I_ (Pbn2_1_) with ≈29 % mixed Li/Co occupation at 4*a* sites. This ratio of cationic mixing is significantly higher than ≈9 % reported for pure β_I_ polymorph,^[13]^ where the relative intensity of reflection 111 is higher and comparable to 220. The minor phase is γ_0_ polymorph (*P*2_1_/*n*) with half of tetrahedra aligned oppositely with respect to that in the β_I_ polymorph. Because of the similarities in the lattice and topology between the two polymorphs, only the major polymorph Pbn2_1_ is used to track the phase evolution in following sections.

First four charge and discharge curves of a Mn‐LCSO cathode are shown in Figure 1 c. The initial charge capacity is 273 mAh g^−1^, indicating 1.68 Li^+^ extraction per formula unit (derived based on a one‐to‐one correspondence between electron flow and Li^+^ extraction and assuming 162.5 mAh g^−1^ for one Li^+^ extracted). The initial discharge capacity is 198 mAh g^−1^, corresponding to ≈1.22 Li^+^ reinsertion, a value nearly doubled from Li_2_CoSiO_4_ in literature.^[14]^ As shown in Figure S2, a small amount of Mn improves the reversibility of lithium chemistry with the same electrochemical characters of Li_2_CoSiO_4_. The second discharge capacity is slightly higher than the initial one with 201 mAh g^−1^, and its voltage profile is very similar to the first one. This is in contrast to Li‐rich and Mn‐rich oxides with oxygen redox (such as O2‐Li_2_MnO_3_,^[8]^ Li_1.2_Ni_0.152_Co_0.1_Mn_0.55_O_2_,^[15]^), Li_3_IrO_4_,^[16]^ Li_5_FeO_4_,^[2]^ and silicates Li_2_FeSiO_4_ and Li_2_MnSiO_4,_ ^[17]^ They all suffer from significant voltage hysteresis with instable discharge plateau below 4 V. The following two cycles repeat well the second cycle in both voltage profile and reversible capacity. No obvious drop in the voltage plateau occurs in the cycling of Mn‐LCSO.

**Figure 2:**
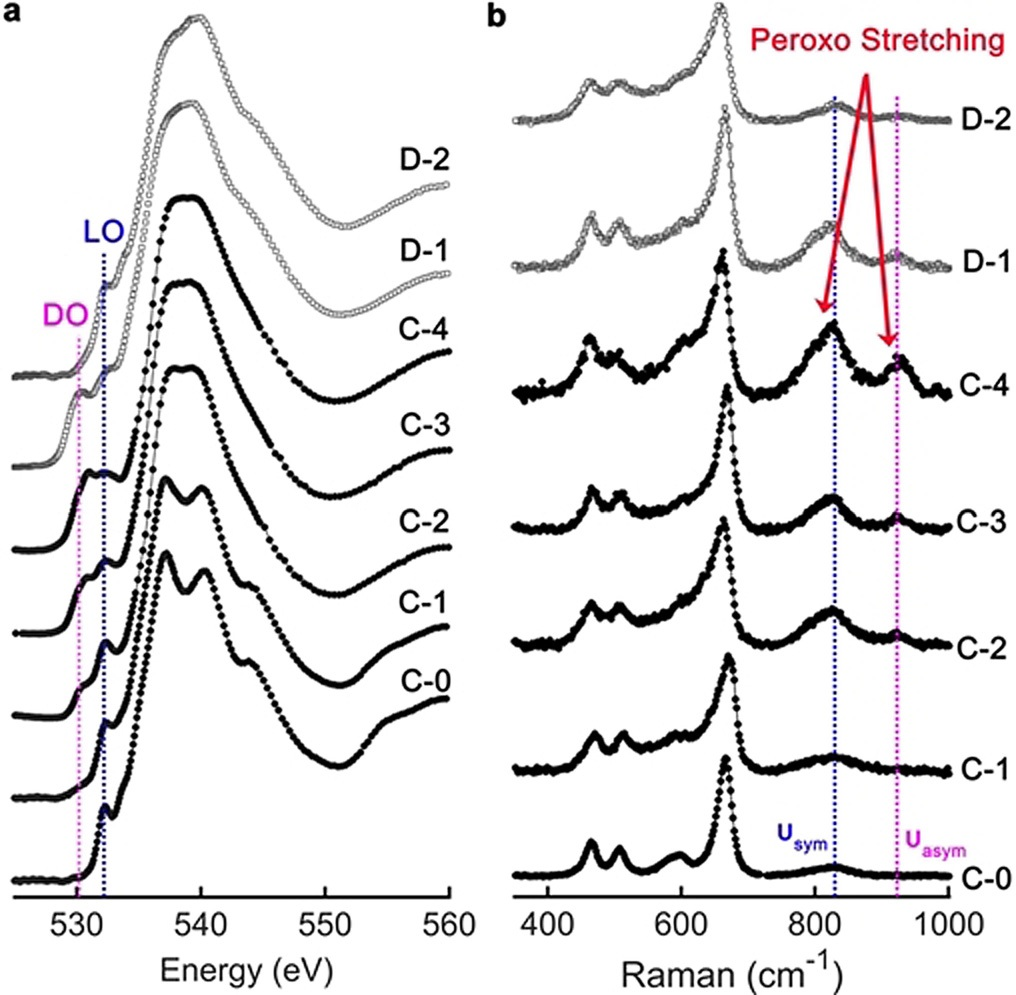
Ex‐situ spectroscopic signatures of oxygen redox in Mn‐LCSO. a) Evolution of synchrotron O K‐edge XANES (TEY) with charging (C‐0 to C‐4) and discharging (D‐1 and D‐2). b) Evolution of Raman spectra at the same charging and discharging states.

Oxygen redox is studied for samples in the first cycle, c.f., Figure 1 c, by ex‐situ synchrotron O K‐edge XANES and Raman spectroscopy. In pristine Mn‐LCSO (C‐0), only a single pre‐edge peak locates at ca. 532.3 eV in the O‐K edge, labelled as the LO in Figure 2 a, which originates from the excitation of O‐1s electron to the O‐2*p* orbitals hybridized with the half‐empty *t_2_* orbitals of the high‐spin Co^2+^ ion in tetrahedral group CoO_4_. During the charging process, the LO peak does not attenuate but broadens. That is consistent with first principles calculation shown in Figure S3a that the contribution from the Co^3+^‐*e_t_* and Co^3+^‐*t_2_* holes are unresolvable and giving a single broad peak, c.f. Figure S3b, which could also be hardly distinguished from the peak from Co^2+^‐*t_2_* hole at ≈0.9 eV higher. The contributions from two subsets are unresolvable as one single broad pre‐edge peak, a spectral feature that is also observed for high‐spin octahedral Fe^2+^ ion in FeO with *e_g_* and *t_2g_* holes.^[18]^ The contributions, however, are different from those in the case of low‐spin Co^4+^‐*t_2g_* hole in delithiated LiCoO_2_, where the contributions from the two subsets can be clearly resolved.^[18a]^ At C‐2, a new pre‐edge peak, denoted as the DO peak, emerges at ca. 530.3 eV, about 2.0 eV lower than the pristine LO peak. The intensity of the DO peak increases significantly and becomes comparable to that of LO peak at C‐3. The DO peak is not from side‐products such as Li_2_CO_3_ or Li_2_O with pre‐edge peaks at ca. 533.9 and 534.1 eV, respectively.^[19]^ It is close to the pre‐edge peak of Li_2_O_2_ at ≈530.5 eV, but higher than the first peak of superoxide at ≈529.0 eV and lower than the first pre‐edge peak of molecular O_2_ at ≈531.0 eV.^[20]^ In addition, the O‐K edge of total fluorescence yield (TFY) mode agrees with the total electron yield (TEY) mode, c.f. Figure S4, suggesting this DO peak comes from peroxo species in the bulk, which compensates to Li^+^ extraction at high‐voltage. During the discharging, the DO peak attenuates significantly from D‐1 to a very weak peak at D‐2, indicating the oxygen species has been largely reduced back to the metal‐oxo ligand at the end of the first Li^+^ re‐intercalation. This confirms the reversibility of the oxygen redox, agreeing with the four stable electrochemical cycles.

Figure 2 b shows the evolution of Raman spectra in the range from 400 to 1000 cm^−1^. For pristine C‐0, the band around 825 cm^−1^, labeled as υ‐sym, is assigned to the symmetric stretching mode of SiO_4,_ while the asymmetric stretching mode is weakly Raman‐active and hardly visible. The other bands at lower frequencies are coupled vibrational modes of CoO_4_ and LiO_4_ with the bending modes of SiO_4_.^[21]^ During the charging process, the intensity of the υ‐sym band increases significantly and a new band emerges at ≈923 cm^−1^, which is close to the frequency of the asymmetric stretching mode of SiO_4_ and labeled as υ‐asym. Both υ‐sym and υ‐asym match with peroxo stretching modes in the range from 800 to 1000 cm^−1^.^[22]^ The occurring of two Raman bands hints the strong coupling of peroxo moiety with SiO_4_, c.f. two possible combined vibration modes shown in Figure S5. This presence of a peroxo bond involving with two vibrational modes is similar to the combination of the W‐O stretching mode with O‐O stretching mode in delithiated Li_4.15_Ni_0.85_WO_6_ giving two bands at 890 and 930 cm^−1^ as suggested by DFT calculation,^[23]^ while different from single peroxo stretching mode measured with shell‐isolated nanoparticle‐enhanced Raman spectroscopy for Li‐rich and Mn‐rich oxides.^[24]^ For Li_4.15−*x*_Ni_0.85_WO_6_, the DFT calculation suggested peroxo stretching dominates the band at 930 cm^−1^, whereas here the Si−O bond is stronger than the O−O bond within the peroxo moiety and thus it could dominate the combined bands. The early emergence of the DO peak and peroxo stretching mode during the charging process suggests the overlap of anionic redox with cationic redox, which could be tightly correlated to heterogeneous delithiation due to low Li^+^ kinetics, similar to the overlap of oxygen redox with Ni^2+^/Ni^3+^ redox in Li_4.15_Ni_0.85_WO_6_.^[23]^

In the discharging, the intensity of υ‐sym and υ‐asym decreases in the same trend as the DO peak evolution, suggesting the density of peroxo moiety decreases in the discharging, too. A sizable amount of peroxo O−O bonds have cleaved in the oxygen reduction reaction. Note, the intensities of both the DO peak and two Raman bands are almost unchanged from C‐4 (at the end of charging) and D‐1 (at the just start of discharging) implies the high stability of peroxo moiety, not inclined to transform to O_2_ molecules. That is different from the formation of molecular O_2_ suggested by the fine structure of RIXS of Na_0.75_[Li_0.25_Mn_0.75_]O_2_, Na_0.6_[Li_0.2_Mn_0.8_]O_2_ and Li_1.2_Ni_0.3_Mn_0.54_O_2_ with increasement at ≈531.0 eV in oxygen‐K absorption edge.^[7a]^ Thus, the two Raman‐active bands concurrent with the DO pre‐edge peak suggest the peroxo formation in the bulk of silicate cathodes.

**Figure 3:**
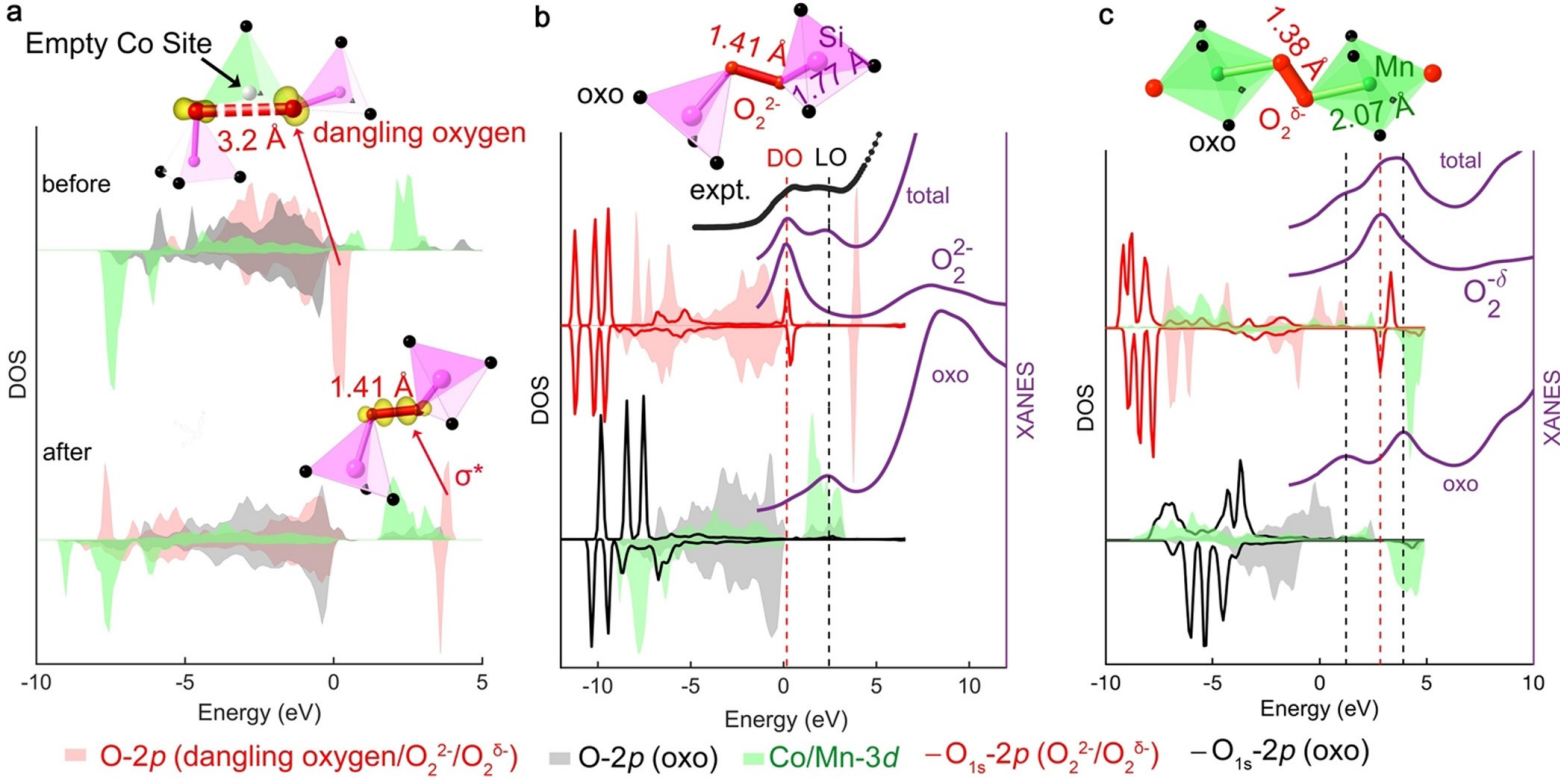
a) First‐principles density of states (DOS) of Co/Li‐site exchanged CoSiO_4_ before (top) and after (bottom) structural relaxation with lithium removal. Charge isosurface is plotted in yellow for oxygen electrons in the indicated peak range. b,c) First‐principles calculations of O K‐edge XANES for systems with the O‐O dimer, and site‐decomposed contributions from metal‐oxo ligand and O_2_^δ−^. b) CoSiO_4_ with peroxo O_2_^2−^ moiety bridging Si ions, “expt” for experimental C‐4 of Figure 2 a. c) Li_5/4_MnO_3_ with O_2_^*δ*−^ (1<*δ*<2) moiety bound to Mn ions. The label “O_1s_” indicates the core relaxation after the 1s electron excitation and “oxo” for lattice oxygen ion. The partial DOS of Co/Mn‐*d* is scaled down by 0.4. The XANES contributions from O_2_^2−^ and O_2_^*δ*−^ are scaled down by 0.2 and 0.5, respectively.

To understand the oxygen transformation path in peroxo formation and validate the spectroscopic signals of the peroxo species formed in the bulk, first‐principles calculations are performed for structural transformation and oxygen‐K XANES. To simplify the modeling without loss of generality, here the focus is on fully delithiated CoSiO_4_ that theoretically corresponds to the highest oxidation state of the material. Four kinds of □_Li_/Co occupation models are considered based on analysis of synchrotron XRD (c.f. Supplemental note 2 and Table S2). Due to the Li/Co mixed occupations, there exist pairs of lattice oxygen ions decoordinated from Co ions, denoted as dangling oxygen ions, similar to the case of Li_2−*x*_MnO_3_.^[6]^ The upper inset of Figure 3 a shows an example of a dangling oxygen pair separated by 3.2 Å, which contain localized unpaired non‐bonding O‐*p* character on the Fermi level, c.f., the charge density isosurface in the plotting of Figure 3 a. After structural relaxation, this dangling oxygen pair forms a peroxo moiety, c.f., the lower inset of Figure 3 a. The peroxo bond formation reopens the gap to stabilize the electronic structure. In contrast, in the model without Li/Co mixed occupation, shown in Figure S6, only metal‐oxo ligand orbitals participate in the electron reorganization similar to LiCoSiO_4_ with one‐Li^+^ extracted.^[11]^ The Co−O bond shrinks from 1.88–1.98 to 1.77–1.81 Å after relaxation. *The electron reorganization in structural relaxation indicates the character of depleted electrons near the Fermi level by Li* ^*+*^ *extraction is the physical root of oxygen transformation*. If the regular metal‐oxo ligand covalence is decoupled by cationic disorder, the non‐bonding O‐*p* electron will be trapped on the dangling oxygen that becomes a highly active free radical‐like moiety prone to dimerize.

The inset of Figure 3 b shows the representative end‐on/end‐on (μ‐η^1^:η^1^‐O_2_) peroxo moiety bridging two SiO_4_ among the nine patterns shown in Figure S7. The Si‐O_2_^2−^ bond is elongated to 1.77 Å with respect to the normal Si‐O *sp* ^*3*^ bond of 1.65 Å. The end‐on/end‐on binding mode had also been reported for Li_0.5_Ir_0.75_Sn_0.25_O_3_,^[5]^ Li_5/4_MnO_3_ and Li_3/4_MnO_3._ ^[6]^ As shown in the inset of Figure 3 c, the Mn‐O_2_^*δ*−^ bond length of 2.07 Å, elongated from Mn−O bond of ca. 1.92 Å, indicates a much weaker binding with the dimer. The strength to bind the dimer depends on the covalence between the binding cation and the dimer, which follows the order: *p‐p* hybridization>*p*‐*d* hybridization. With pure *p*‐*p* hybridization, the O‐O dimer species is expected to be more strongly bound by Si, comparing to Sn or Ir/Ru in Li_0.5_Ir_0.75_Sn_0.25_O_3_ ^[5]^ and Li_2_Ru_1−*y*_Sn_*y*_O_3_ ^[1a]^ with *p*‐*p* hybridization mixed with *p*‐*d* hybridization in the binding, and Mn/Co/Ni in Li‐rich and Mn‐rich oxides with *p*‐*d* hybridization. The strong covalence of Si‐O_2_^2−^ bond could lock the O‐O dimer in the oxidation state of peroxo moiety, instead of forming mononuclear superoxo moiety or molecular O_2_, and the peroxo moiety can be strongly confined in the bulk even at high‐voltage for cumulative cationic and anionic redox. This is supported by the enhanced capacity in our samples that contain a high degree of cationic disorder with 29 % Li/Co mixed occupation. Such a high cationic disorder is always avoided in oxides like Li(NiCoMn)_1/3_O_2_.^[25]^ But our samples deliver a reversible capacity, nearly doubled from that of cationic ordered Li_2_CoSiO_4_.[^14b^, ^26^] The improvement could be largely attributed to the peroxo formation via dangling oxygen occurring with Li^+^ kinetics in virtue of cationic disorder but not Co or Si migrations during the charging process as Mn/Co/Ni and Sn/Ir migration in Li‐rich and Mn‐rich oxides^[9]^ and Li_2_Ir_0.75_Sn_0.25_O_3_,^[5]^ respectively.

As shown in Figure 3 a, the peroxo moiety contains a pair of unoccupied anti‐bonding σ*. That is the common feature of all O‐O dimers regardless of their oxidation states, in contrast to all metal‐oxo ligands including the terminal‐oxo ligands that do not have this orbital character. This distinction is the base to differentiate lattice and oxidized oxygen in X‐ray spectroscopies. First‐principles calculations of O K‐edge XANES are performed for CoSiO_4_ with peroxo models presented in this work. Site‐decomposed contributions from metal‐oxo ligand and O_2_^2−^ are shown in Figure 3 b. We include Li_5/4_MnO_3_ (Figure 3 c) to generalize our theoretical study. In CoSiO_4_, there is one single oxo pre‐edge peak, whereas in Li_5/4_MnO_3_, there are two oxo pre‐edge peaks with a gap of ca. 2.5 eV associated with the unoccupied spin‐up and spin‐down Mn‐*3d* states, respectively. In both peroxo and superoxo, when the O‐*1s* electron is excited to the σ^*^ orbital which is unoccupied in both species, a pre‐edge peak is generated with much higher intensity than in metal‐oxo due to high localization. The excitation energy increases with the oxidation state of the species, analogue to the trend in alkali‐peroxide/superoxide.^[20c]^ As shown in Figure 3 b, due to the core relaxation with the O‐*1s* electron excitation, the σ* states in CoSiO_4_ are moved downward below the unoccupied Co‐*3d* states, resulting in the signature pre‐edge peak ≈2.0 eV below the oxo peak. Thus, the two sets of pre‐edge peaks from the lattice metal‐oxo ligand and peroxo moiety O_2_^2−^ can be easily distinguished in the spectra. Their relative positions agree very well with the experimental O K‐edge XANES spectroscopy shown in C‐4 of Figure 2 a, confirming the DO signature is from the excitation of the O‐*1s* electron to the σ* orbital of the dinuclear peroxo moiety formed in the bulk amid oxygen redox. A further comparison is provided for Li_1/2_CoSiO_4_ in Figure S8, validating the signature over the delithiation range.

It is clear that the transition from the O‐*1s* electron to the σ* orbital of the dimer is independent of the details of the dimers. The O‐O dimer of Li_5/4_MnO_3_ shown in Figure 3 c is an intermediate dimer between superoxo and peroxo with a small fraction of spin‐up π* states unoccupied. Its σ* orbital is located above the spin‐up Mn‐*e_g_* states, giving a signal peak between the two Mn‐oxo peaks. This pattern is close to the enhancement on the right shoulder of the first oxo peak observed in Mn‐based alkali‐rich oxide cathodes.[^3a^, ^7a^] Surprisingly, when the O‐*1s* electron is excited to the unoccupied π* states, the signal is hardly observable, due to the strong back‐bonding with Mn ions. This is different from the strong peak given by the unoccupied π* states in either standalone alkali‐superoxides or oxygen molecule.^[20c]^ Thus, for all O‐O dimers in the bulk, the transition from O‐*1s* to the σ* orbital is a detectable signature linked to its redox pathway. This is a reliable methodical characterization to discern the lattice oxygen transformation. The signals given by the σ* orbitals in XAS and RIXS amid oxygen redox may be observable when the peroxo/superoxo species are confined in the bulk.

**Figure 4:**
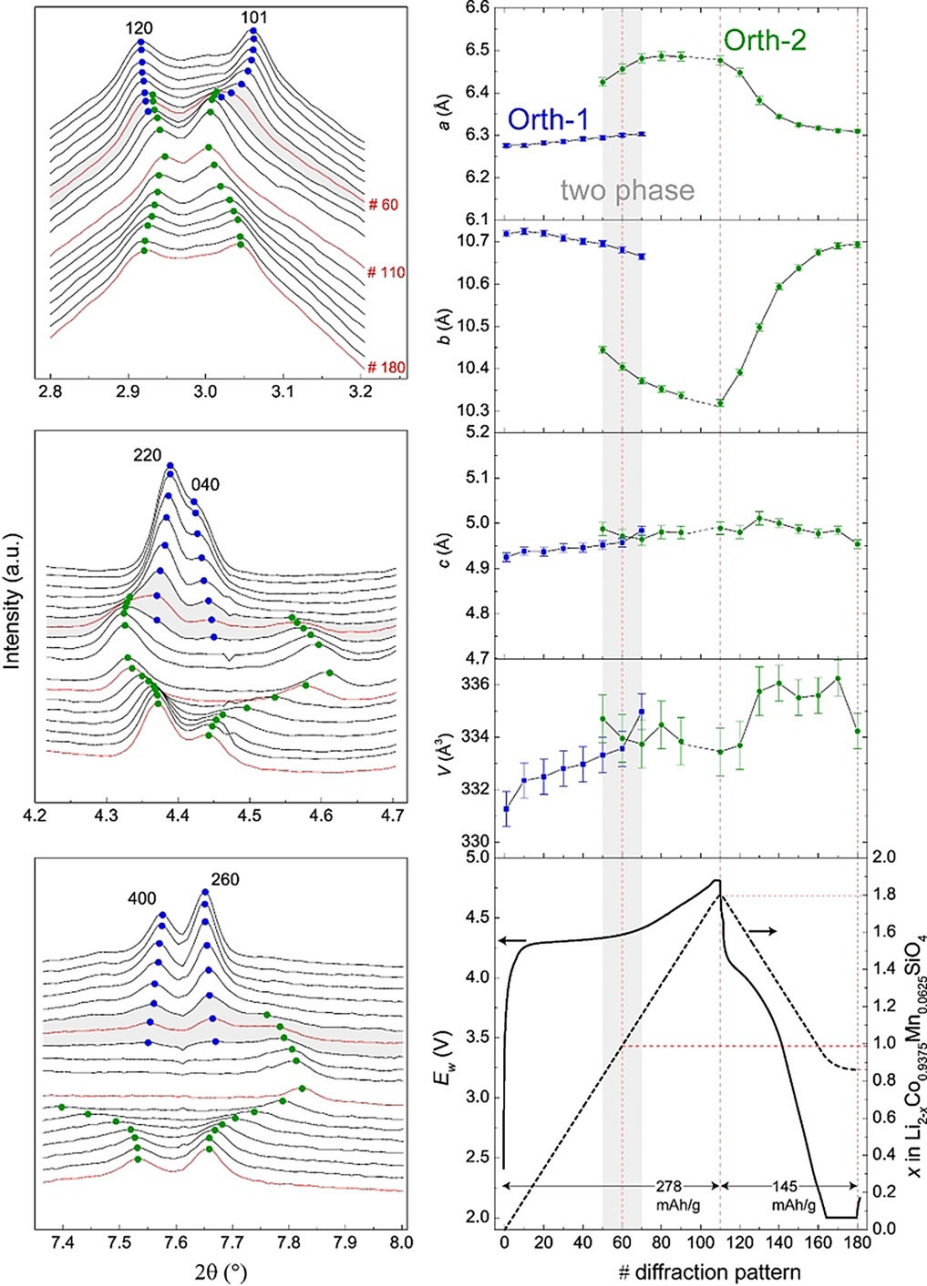
Operando synchrotron XRD plots for Mn‐LCSO. a) Sections of background‐corrected diffraction patterns (*λ*=0.20729 Å). b) Fitted lattice parameters *a*, *b*, *c* and cell volume *V* and the corresponding charging–discharging curves between 2.0–4.8 V. The dotted lines correspond to a beam loss, with no patterns recorded from #93 to #103. The whole pattern of the in situ battery cell setup is shown in Figure S9.

The lattice evolution of Mn‐LCSO during the first cycle between 2.0 V and 4.8 V vs. Li anode is examined by operando synchrotron XRD, as shown in Figure 4. Here the orthorhombic lattice is used to track the structural evolution. As shown by the blue dots labelled as Orth‐1 phase in Figure 4 a and 4b, up to pattern # 40, which corresponds to ≈0.65 Li^+^ extraction with very little oxygen oxidation, the reflections of Orth‐1 phase shift only slightly, with the lattice changing slightly and smoothly, corresponding to a very flat charging plateau, typically associated with cationic redox, i.e., Co^2+/^Co^3+^. The intensity is reduced significantly for the high‐indexed reflections such as 220 and 260, but the intensity reduction is small for the low‐indexed reflections, c.f. Figure S10, suggesting a disruption of structural details on the long‐range correlation length even occurring at the early delithiation stage. This behavior is different from the continuous solid‐solution reaction in layered oxide Li(NiCoMn)_1/3_O_2_,^[25]^ and the abrupt two‐phase reaction in LiFePO_4_.^[27]^ In the ensuing delithiation, a set of new reflections stem from another orthorhombic phase Orth‐2, shown by the green dots in Figure 4 a and b. The Orth‐2 phase becomes the only lattice at # 80, which corresponds to ≈1.3 Li^+^ extraction, remarkably coinciding with the major density of peroxo moiety. The lattice transition from Orth‐1 phase to Orth‐2 phase presents a stepwise change in *a* and *b* axes, but almost no change for *c* axis. The expansion of *a*, ca. 3.2 %, is almost compensated by the contraction of *b*, ca. 3.0 %, thus the volume of the lattice is almost unchanged from Orth‐1 to Orth‐2. This is very contrary to the 16.9 % expansion from Li_2_CoSiO_4_ to CoSiO_4_ predicted for the Co^2+^/Co^4+^ cationic redox by first‐principles calculations.^[12]^ Thus, the lattice evolution suggests that Orth‐2 phase does not involve Co^4+^O_4_. The phase coexistence of Orth‐1 and Orth‐2 from about # 50 to # 70 points to a classification as the type of first order phase transition even though there is almost no volume change of the unit cell. This is surprising but understandable, considering the dimerization that occurs in the Li‐extracted empty volume. The phase coexistence indicates the dimerization reduces the volume expansion, peroxo formation counters the electrostatic repulsion from oxidized metal‐oxo ligands. That should be very benign to suppress the phase transition and structural collapse of the delithiated host. When the Co^2+^/Co^3+^ approaches finishing near LiCoSiO_4_, only peroxo formation is active in the high‐voltage region. The reflections of the Orth‐2 phase show larger shifts than the Orth‐1 phase, corresponding to a rising charging profile.

During the discharging process, the reflections continuously shift back to those values of the charged Orth‐1 phase with a pronounced broadening of the reflections around # 130, which corresponds to ca. 0.3 Li^+^ reinsertion. The lattice of the Orth‐2 phase at the end of discharging only slightly deviates from that of the Orth‐1 phase at the end of charging, indicating the Orth‐2 phase with Li^+^ reinserted is very close to the flat Orth‐1 phase. The reversibility in structural evolution mitigates voltage hysteresis that often occurs with oxygen redox,[^7a^, ^28^] and here contributes to those similar voltage profiles in the four cycles. The reversibility could benefit from the high Li/Co mixed occupation that does not necessitate Co migration to facilitate peroxo formation,^[6]^ and strong Si‐peroxo binding. However, the intensity of reflections at the end of discharge is much lower than at the beginning of charge, suggesting the disruption of the long‐range structural details has not been fully recovered. The incomplete reversibility in structural evolution and oxygen redox combined with intrinsically low conductivity of silicate cathode may contribute to the large irreversible loss of capacity. In addition, it is also proposed that the kinetics associated with oxygen redox could be intrinsically low.^[9]^ Its origin may be due to the insulating nature of the dimer in general. The low electric conductivity originated from silicate structure and oxygen redox worsen both the rate and cycle performance. Note there is only one type of Orth‐2 phase during discharging. The step‐jumping between Orth‐1 and Orth‐2 in the charging does not reappear in the discharging. The reduction of Co ion may strongly interwind with the O‐O dimer cleavage during the solid‐solution‐like reaction within the Orth‐2 phase. This indicates a smooth merging of cationic and anionic reduction at the same 4 V region, i.e., peroxo species conforms anionic redox with cationic redox in the same voltage region.

**Figure 5:**
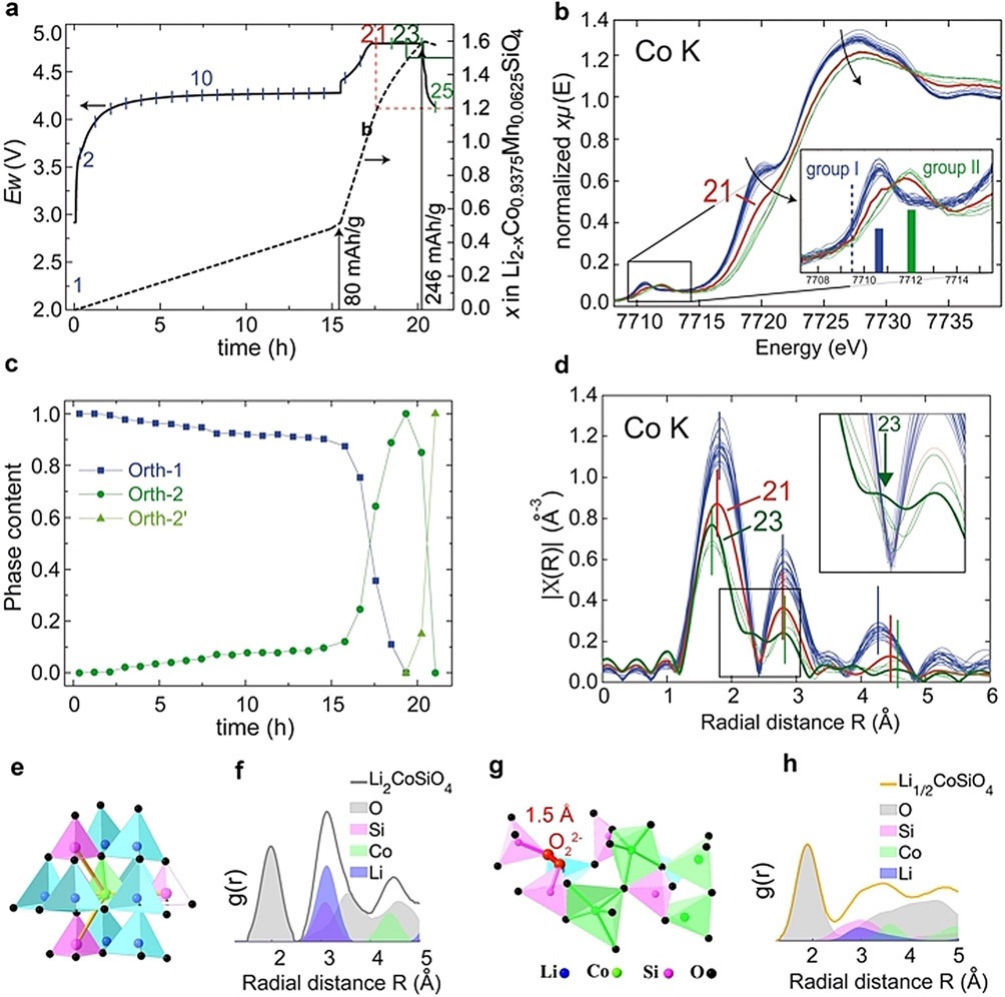
Evolution of medium‐range order. a) Charging curve to 4.8 V for Mn‐LCSO for operando XAS measurement. b) Operando normalized Co K‐edge XANES. c) Phases evolution derived from linear combination fitting (LCF) of XANES obtained by using XAS # 1, # 23 and # 25 as basis representing phases Orth‐1, Orth‐2 and Orth‐2′, respectively. d) EXAFS *k* ^2^ *χ*(*k*) Fourier transform (FT) spectra. Atomistic models and PDF of Co ions with contributions from all elements for e,f) ideal Pbn2_1_‐Li_2_CoSiO_4_ and g,h) Li_1/2_CoSiO_4_ with a peroxo moiety.

The record and analysis of operando synchrotron XAS of Co K‐edge with charging up to ≈1.5 Li^+^ extraction are shown in Figure 5 a–d. The XANES can be grouped into two eminent sets: group I with blue lines # 1–20 and group II for green lines # 22–25. The pre‐edge peak shifts and broadens from group I to group II. That indicates oxidation of Co^2+^ to Co^3+^, consistent with the slightly shift and small broadening of LO peak in Figure 2 a. The shoulder at 7720 eV gradually decreases within Group I and disappears in group II. The Co−O bond length remains almost unchanged at ≈1.97(2) Å for group I as shown by the first peak of the Fourier transformation (FT) of extended X‐ray absorption fine structure (EXAFS), in line with the only slightly changing lattice parameters of Orth‐1. The Co−O bond length reduces to ≈1.88(1) Å in group II, in line with Co^3+^−O bond but not complying with the Co^4+^−O bond length of ≈1.77–1.81 Å as suggested from DFT calculations. The red line #21 in Figure 5 b shows mixed feature of group I and II in both main edge and pre‐edge, with the dominant phase flipped to be the Orth‐2 phase as suggested by the LCF in Figure 5 c. That is in line with the end of the two‐phase region and the beginning of the single Orth‐2 phase at in‐between pattern # 70 and # 80 of the operando synchrotron XRD. There is no obvious change in XANES within group II, indicating no further Co oxidation after ≈1.3 Li^+^ extraction. That suggests oxygen oxidation will compensate the following Li^+^ extraction and oxygen oxidation is mixed with Co oxidation in early Li^+^ extraction, consistent with early emergence of the DO peak and peroxo stretching mode.

Within group II, the medium coordination environment of Co suffers prominent distortion. At XAS # 23, corresponding to ≈1.48 Li^+^ extraction, the rising valley evolves to a plateau between the first and second peaks of the FT of EXAFS, accompanied by the attenuation of the third and fourth peaks, indicating medium‐ to long‐range distortion of coordination environments for Co ions and disruption of the long‐range order. That plateau decreases and returns to a valley for XAS # 24 and # 25, suggesting that the medium‐range distortion is largely restored at the end of the charging process. The medium‐range coordination of Co ions is analyzed by simulated atomistic pair density functions (PDF) for pristine Li_2_CoSiO_4_ and Li_1/2_CoSiO_4_ with a peroxo moiety based on first‐principles models, c.f. Figure 5 e–h. As shown, Co−O bond contributes to the first peak of PDF. Due to corner sharing of each CoO_4_ with one SiO_4_ and two LiO_4_, Co‐Si and Co‐Li distance are about 3.1 Å (Co‐Si 3.07–3.10 Å, Co‐Li 3.09–3.19 Å, respectively), contributing to the second peak of PDF for the pristine Pbn2_1_‐Li_2_CoSiO_4_. Figure 5 g shows a typical dinuclear Si‐peroxo moiety in Li_1/2_CoSiO_4_, which theoretically corresponds to the delithiated phase of XAS # 23. The oxygen sublattice distorts and two of the four Co ions deviate from the pristine tetrahedral center and form CoO_5_ trigonal bipyramids, each sharing one edge, rather than a corner with the neighboring SiO_4_, and the Si‐Co distance decreases to ≈2.60 Å (2.58 and 2.61 Å). These Si ions give rise to the valley between the first and second coordination shell of Co ions in the PDF. Simultaneously, some Co−O bonds elongate, e.g., to 2.37 Å, and those O ions are also located in the same intermediate range of the Co ion. Thus, a plateau appears between the first and second peak in the PDF as shown in Figure 5 h, reproducing the medium‐range distortion captured by operando Co‐XAS # 23, as shown in Figure 5 d. From the model construction of Figure 5 g, we find that the Co needs to start from an octahedral site while it finally relaxes to CoO_5_ trigonal bipyramids. This may imply that Co dynamic migration may happen with accumulation of the peroxo moiety, suggesting the instability of heavy cation ions in the deeply delithiated phase.

## Conclusion

This work uses Li_2_Co_0.9375_Mn_0.0625_SiO_4_ to show an oxygen redox reaction that incurs peroxo moiety. Electronic and vibrational spectroscopies are combined to evidence peroxo moiety in the bulk cathodes. First‐principles calculations unravel electron localization is the driving force for oxygen dimerization in cationic disordered structures. The overall spectroscopic and structural evolution with nearly two Li^+^ extractions can be described by two oxidations, somewhat successive but overlapped with: 1) the cationic oxidation Co^2+^→Co^3+^ dominating the solid‐solution‐like reaction within Orth‐1 phase; 2) anionic oxidation 2 O^2−^→O_2_^2−^ accompanied by the phase transition from Orth‐1 to Orth‐2 and medium‐range distortion within the Orht‐2 phase. Electron localization and intrinsic slow kinetics associated with the O‐O dimer are responsible for the irreversible capacity loss and induced voltage decaying in the cycle. The covalence in the binding mode is the key factor determining oxygen redox pathway. Diverse phenomena of oxygen redox can be understood from their difference in the redox pathway that determines the conformation, stability and oxidation state of oxidized oxygen species in the bulk. Non‐metal elements are suggested as a mediator of covalence to rationally design the redox pathway. The spectroscopic signature arising from the O‐*1s* electron to the σ* orbital of the dimer offers an experimental criterion to resolve the long‐protracted controversy on the chemical entity of oxygen redox reactions in the bulk.

Finally, our study indicates that the peroxo species entails two significant advantages: First, it mitigates oxygen release and structural collapse in the high oxidation states. Second, it conforms the anionic redox with the cationic redox at the same high‐voltage region. Both advantages associated with peroxo species may enable doubling or tripling of the scope of cumulative anionic and cationic redox for high energy‐density cathodes.

## Conflict of interest

The authors declare no conflict of interest.

## Supporting information

As a service to our authors and readers, this journal provides supporting information supplied by the authors. Such materials are peer reviewed and may be re‐organized for online delivery, but are not copy‐edited or typeset. Technical support issues arising from supporting information (other than missing files) should be addressed to the authors.

Supplementary
