# Supplementary material for: Peroxo Species Formed in the Bulk of Silicate Cathodes
Source: Angew Chem Int Ed Engl. 2021 Mar 24;60(18):10056–63. doi: 10.1002/anie.202100730 (PMC8251627; doi:10.1002/anie.202100730)
Supplement: Supplementary file 1 — Supplementary [file ANIE-60-10056-s001.pdf]

Angewandte Chemie
Eine Zeitschrift der Gesellschaft Deutscher Chemiker

Supporting Information

Peroxo Species Formed in the Bulk of Silicate Cathodes

Zhenlian Chen+, Bjoern Schwarz+, Xianhui Zhang+, Wenqiang Du+, Lirong Zheng, Ailing Tian, Ying Zhang, Zhiyong Zhang, Xiao Cheng Zeng,* Zhifeng Zhang, Liyuan Huai, Jinlei Wu, Helmut Ehrenberg, Deyu Wang, and Jun Li**

anie_202100730_sm_miscellaneous_information.pdf

Supplementary Information

This file includes:
Experimental
Supplementary Figure 1-9
Supplementary Table I-II
Supplementary Notes 1-3
Supplementary References

Experimental

Materials synthesis

$Li_2Mn_{0.0625}Co_{0.9375}SiO_4$ was prepared by a hydrothermal reaction as reported in our recent works.[1] In this work, doping source $MnCl_2·4H_2O$ (Aldrich, 99%) was mixed with $CoCl_2·6H_2O$ (Aldrich, 99%) with a molar ratio of 6.25:93.75.

Electrochemical measurements

The working electrodes were fabricated by mixing active material, super P carbon and polyvinylidene fluoride (80:10:10, *w/w/w*) using N-Methyl-pyrrolidone. CR2032 coin cells were assembled with the prepared electrode (14 mm in diameter), lithium foil, and 100 μL electrolyte (1M $LiPF_6$ in EC/DMC, 1:1, *v/v*) in an Ar-filled M-Braun glove box. The electrochemical performance was tested with current densities of 5 mA g^{-1} at 25 °C (Land 2001A, Wuhan, China). The electrode loadings in this part are around ~0.6 mg cm^{-2}.

XRD and Rietveld refinement

Synchrotron XRD patterns of pristine sample were collected in Debye-Scherrer transmission mode (0.5 mm glass capillaries) using the X-ray powder diffraction endstation of BL04-MSPD beamline at ALBA synchrotron (Barcelona, Spain) Wavelength: 0.41311(1) Å. Operando synchrotron XRD was performed at the synchrotron facility PETRAIII/DESY (Hamburg, Germany) at beamline P02.1 in transmission mode. A fast area detector PerkinElmer XRD1621 with 2048 × 2048 pixels (200 μm^2 size) was used as flat panel detector and the wavelength was 0.20729(1) Å. The electrochemical tests used custom made in-situ coin cells with Kapton windows (5 mm hole), 1 piece of Whatman glass fibre as separator, ~150 µL of Merck LP30 as electrolyte (1 M $LiPF_6$ / EC-DMC (1:1 *wt.*%) and 200 µm Li foil as counter electrode. EC sequence: Constant current (CC) charge with 50/3 mA g^{-1} to 4.8 V; Constant voltage (CV) at 4.8 V for 1 h; OCV period for 15 min; CC discharge with 50/3 mA g^{-1} to 2.0 V; CV at 2.0 V for 2.5 h. The overall time is about 30 h. It is worth noting that the electrode loadings in this part are around ~2.65 mg cm^{-2}.

The GSAS software package[2] was used to perform Rietveld refinement, where variation in mixed Li/Co (Mn) occupation was allowed.

XAS measurements

Operando synchrotron Co-K edge X-ray absorption fine structure (XAFS) measurement was performed at beamline P64 (Petra III/DESY, Hamburg, Germany). Cell set-up as for EC laboratory measurements but assembled with custom made in situ coin cells with Kapton windows for beam penetration. For electrochemical tests, in situ coin cells 2032 with 12 mm cathode sheet, 1 piece of Whatman separator, Merck LP30 electrolyte, and 200 µm Li foil as counter electrode were prepared in Ar atmosphere. EC sequence galvanostatic charging with 5 mA/g for 15 h and then the current densities were enhanced to 50 mA/g until charge to 4.8 V with a subsequent constant voltage period. It is worth noting that the electrode loadings in this part are around ~2.65 mg cm^{-2}.

Ex situ soft X-ray absorption structure (sXAS) of O K-edges were firstly collected via total electron yield (TEY) mode at the beamline 4B7B at Beijing Synchrotron Radiation Facility (BSRF), China. O-K edge is also collected using surface-sensitive TEY and bulk-sensitive total fluorescence yield (TFY) modes simultaneously at beamline 02B02 of the SiP·ME^2 platform at the Shanghai Synchrotron Radiation Facility (SSRF). The photon energy of O-K edge was calibrated with the spectra of the reference samples $SrTiO_3$ measured simultaneously.[3]

Data reduction and analysis of XAS were performed by using the ATHENA software.[4]

Ex situ Raman measurement

The Raman measurements were performed on Renishaw inVia Reflex with excitation wavelengths of 532 nm and power density of ~6.1 mW/μm^2. The beam was focused through 50x collection optic with a numerical aperture of 0.75 and spread on the surface to a circular area in diameter of approximately 0.865 µm.

Morphology and EDX

Low-magnification and high-resolution TEM images were taken on a field-emission transmission electron microscope (FEI Tecnai F20) with electron accelerating voltage of 200 keV, and the elements distribution in sample was measured on an energy dispersive X-ray spectroscopy (EDX).

First-principles modelling

Structural relaxations were performed with Vienna Ab initio Simulation Package (VASP 5.4)[5] using the projector augmented wave (PAW) pseudopotential.[6] XANES simulations were performed with using the Quantum-Espresso package and the projector augmented wave method based on ultrasoft pseudopotential.[7] Hubbard correction with the rotationally invariant approach for dealing with the double-counting term based on the PBE functional[8] was adopted for all calculations.

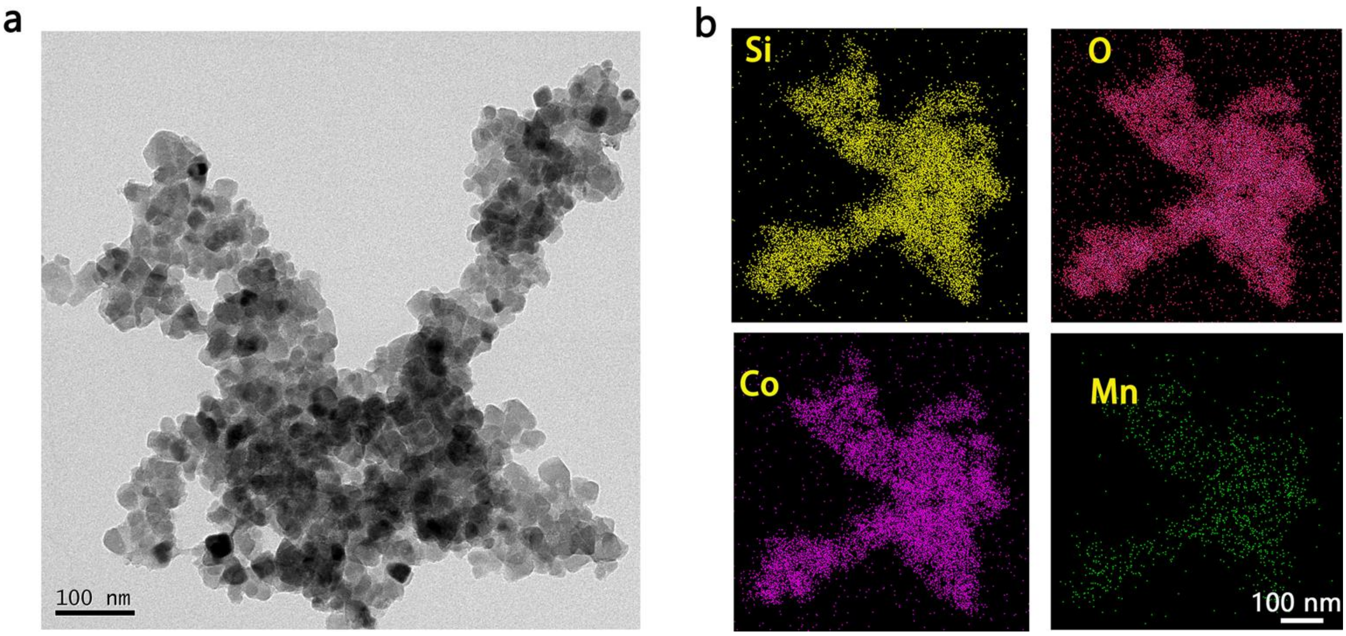

Supplementary Figure 1. a, Low-magnification TEM image. **b**, Scanning transmission electron microscope-energy dispersive x-ray spectroscopy (STEM-EDS) mapping.

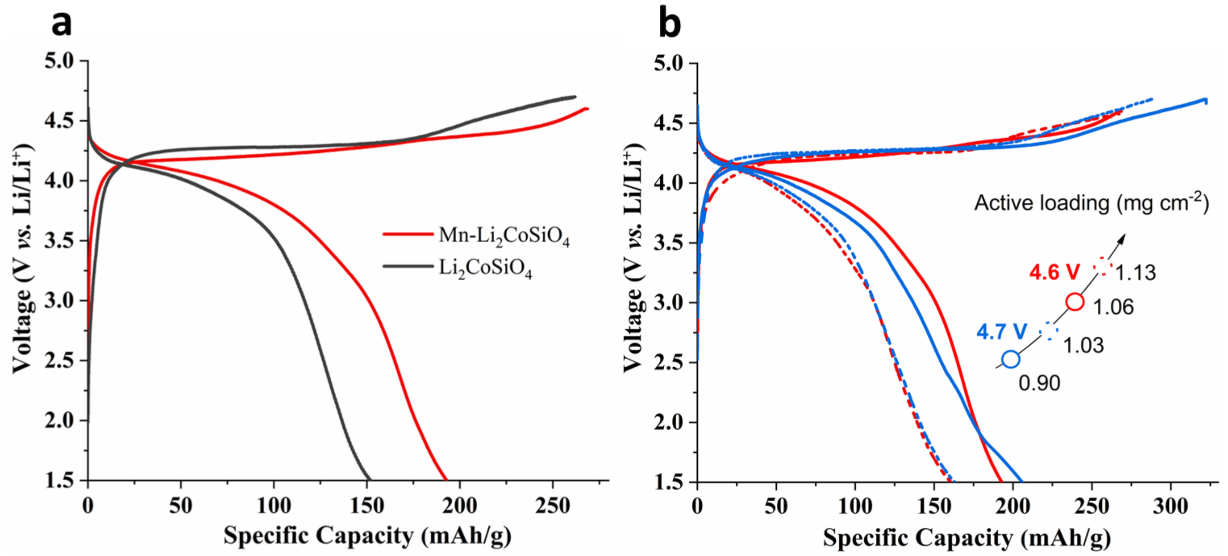

Supplementary Figure 2. Evaluation of the initial charging and discharging characteristics of 6.25% Mn substituted Li_2CoSiO_4 (Mn-Li_2CoSiO_4). a, Comparison between pure Li_2CoSiO_4 and Mn-Li_2CoSiO_4; b) dependence of cathode performance on the Mn-Li_2CoSiO_4 loading with charging up to 4.6 and 4.7V.

Supplementary Note 1

Figure S2 illustrates the significant improvement of the electrochemical performance of Mn-Li_2CoSiO_4. All samples are carbon coated. Figure S2a shows pure Li_2CoSiO_4 delivers charge and discharge capacities at 262.0 and 152.2 mAh/g, respectively, after charging to 4.7 V (with a loading of 1.01 mg cm^{-2}). With Mn substitution, the charging profile agrees very well with the pure Li_2CoSiO_4 sample but the discharge curve has a slightly higher voltage plateau with ~50 mAh/g more reversible capacity, indicating improved overpotentials. The testing sample is charged to 4.6V with a higher loading of 1.06 mg cm^{-2} than that of the pure Li_2CoSiO_4 sample. This indicates the main benefits of a small amount of Mn substitution is to improve the electrochemical performance with a higher value of reversible capacity accessed in the electrochemical cycling.

Due to the intrinsically slow kinetics and low electrical conductivity, the family of Li_2CoSiO_4 suffers from bad rate performance, which shows a strong dependence of charging and discharging capacities on the cathode loadings. Figure S2b shows there is an abrupt decrease in the discharging capacity along with the increase of active loadings, but the variation in charging profiles is small with respect to the charging voltage and active loadings. The threshold of loadings varies slightly with the upper charging voltage. The reversible capacity seems converged at high loadings, and insensitive to the upper voltage limits.

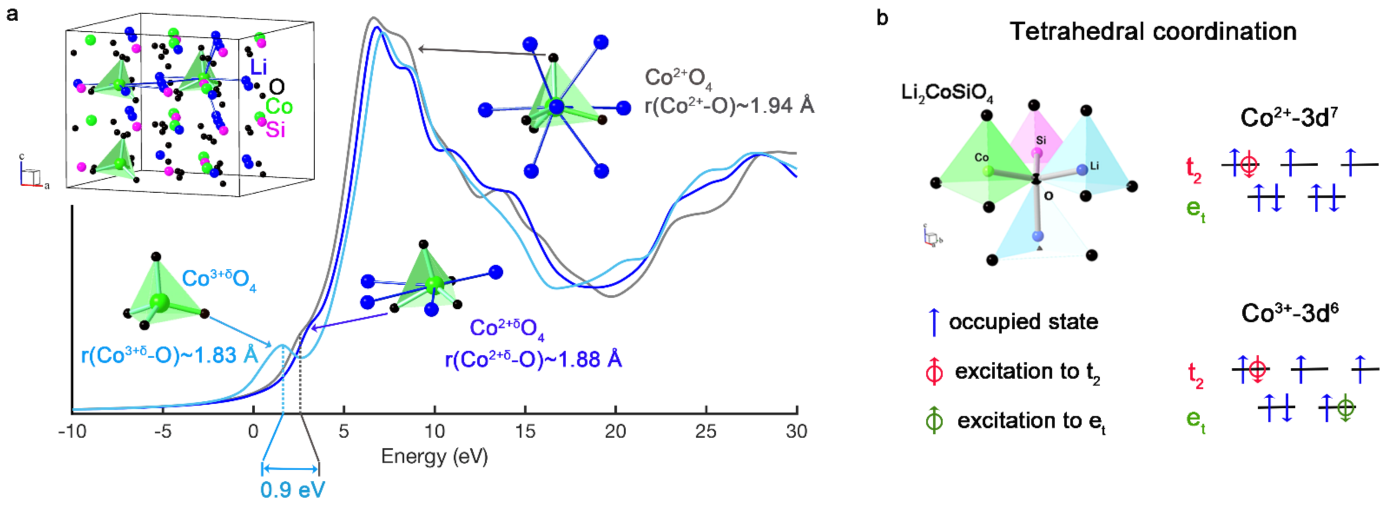

Supplementary Figure 3. The evolution of oxygen-K pre-edge with Co oxidation. **a,** Site-projected XANES of O-K edge for delithiation model **without** dimer formation in $Li_{3/2}CoSiO_4$ from first-principles calculations. In order to understand the evolution of XANES with Co oxidation, we build a model containing 16 f. u. $Li_{3/2}CoSiO_4$, where 8 Li ions are indirectly coordinated to Co^{2+} ion, 4 Li ions to $Co^{2+\delta}$ ion and all the Li ions are removed around $Co^{3+\delta}$ ion. **b**, Tetrahedral coordination in Li_2CoSiO_4 and corresponding electronic configuration of Co^{2+} and Co^{3+} ions. Here, the circle with the arrow indicates the hole hybridized with O-2*p* that accepts the O-*1s* excitation.

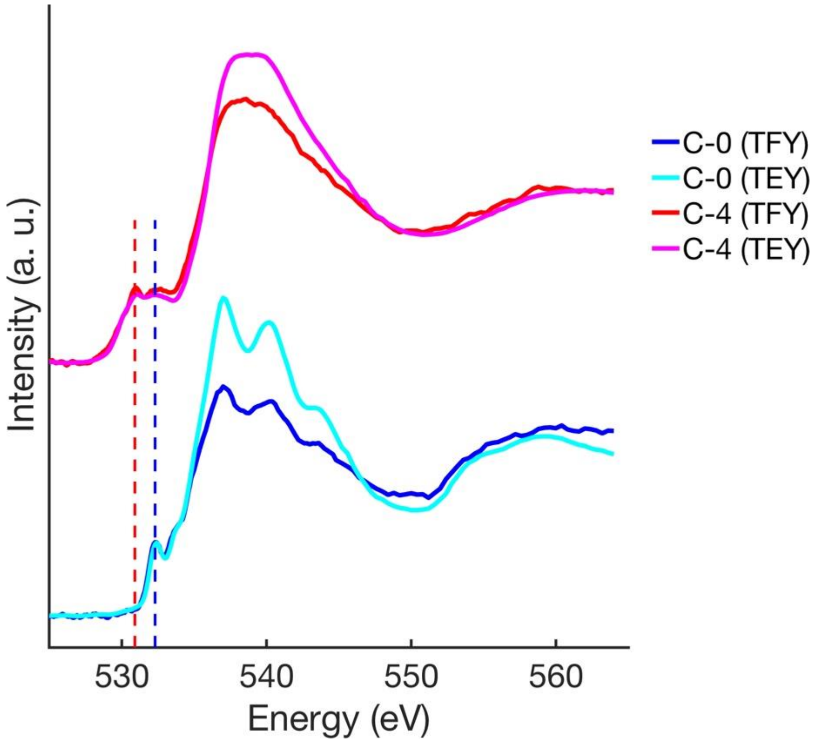

Supplementary Figure 4. Measured XANES of O-K edge with total electron yield (TEY) and total fluorescence yield (TFY) modes simultaneously for Mn-Li_2CoSiO_4 at the beginning (C-0) and end of the charge (C-4), respectively.

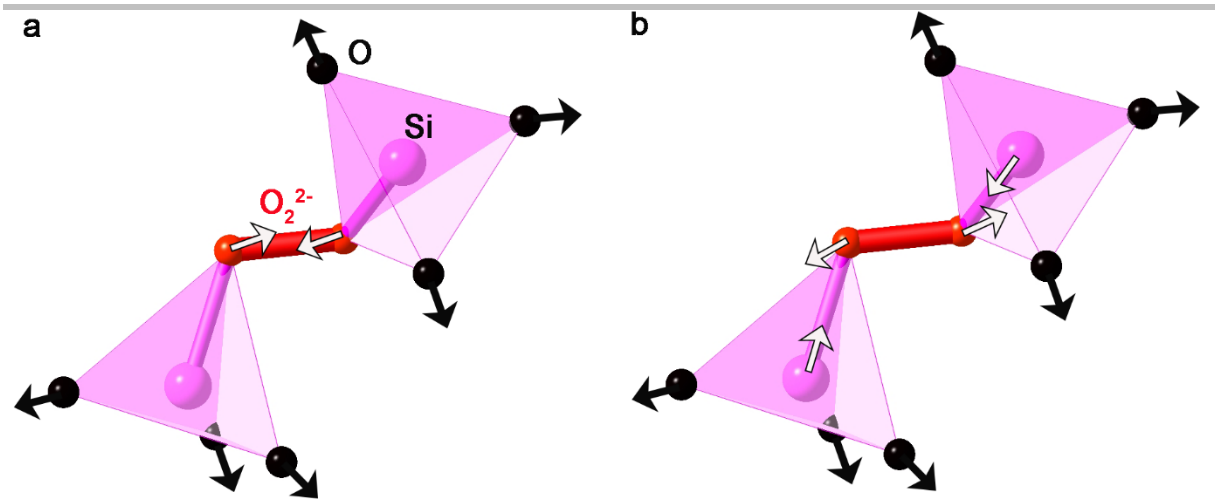

Supplementary Figure 5. The coupling scheme of O-O stretch mode with **a**, symmetric SiO_4 stretch mode and **b**, asymmetric SiO_4 stretch mode.

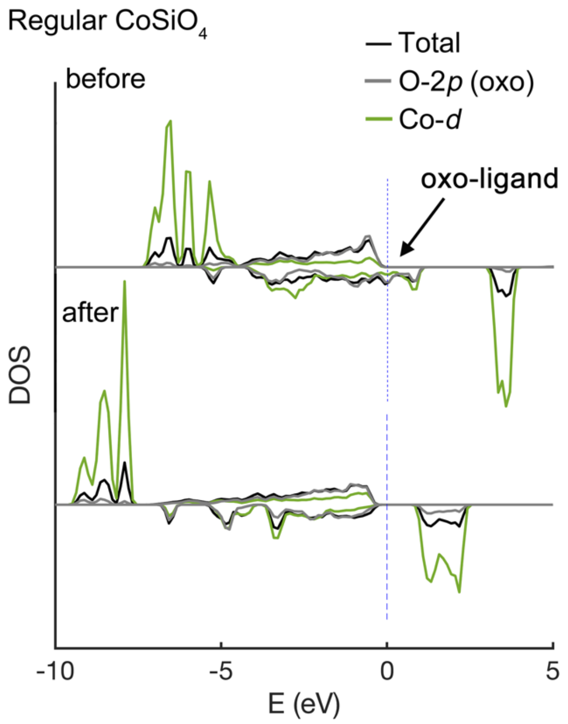

Supplementary Figure 6. First-principles density of states of regular Co/Li-site occupied $CoSiO_4$ between and after structural relaxation with lithium removals.

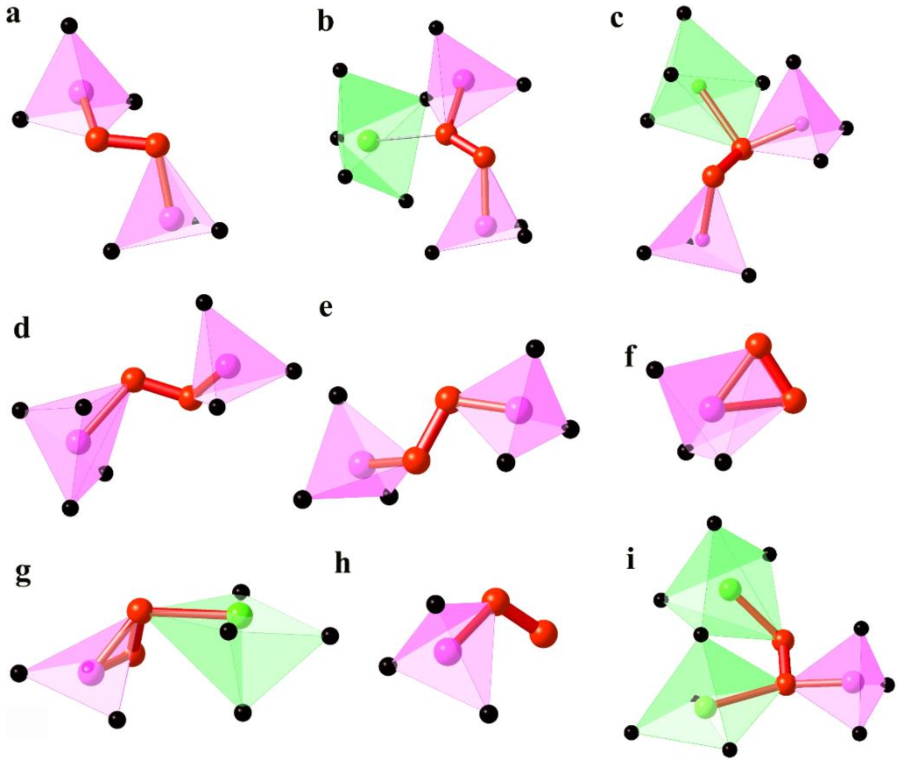

Supplementary Figure 7. Side view of nine patterns of binding of oxygen dimers to Co/Si ions. The dimer moiety is represented by red spheres linked with a red stick. The green, pink, and black spheres represent Co, Si, and lattice oxygen ions, respectively, and the CoO_n and SiO_n polyhedra are represented in green and pink, respectively.

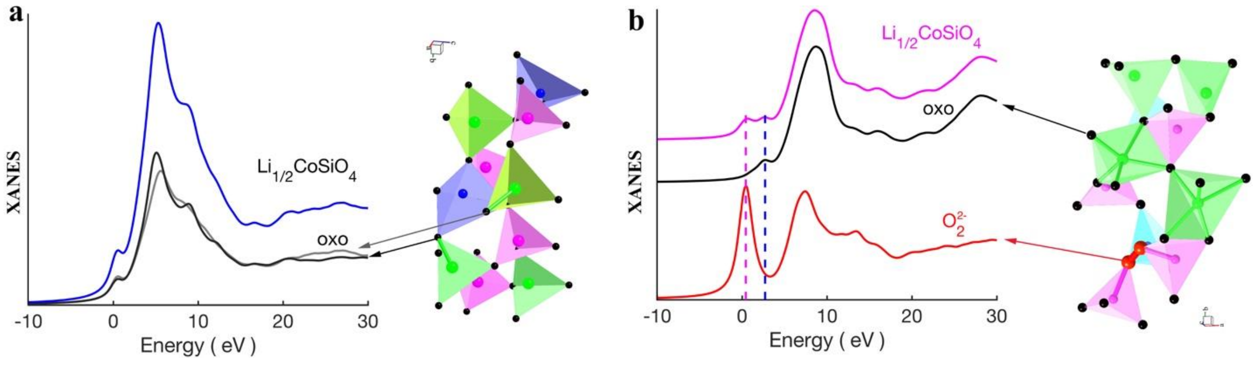

Supplementary Figure 8. Site-projected XANES of O-K edge for $Li_{1/2}CoSiO_4$ from first-principles calculations. a. Conventional delithiation model without dimer formation. b. Delithiation model with peroxo dimer.

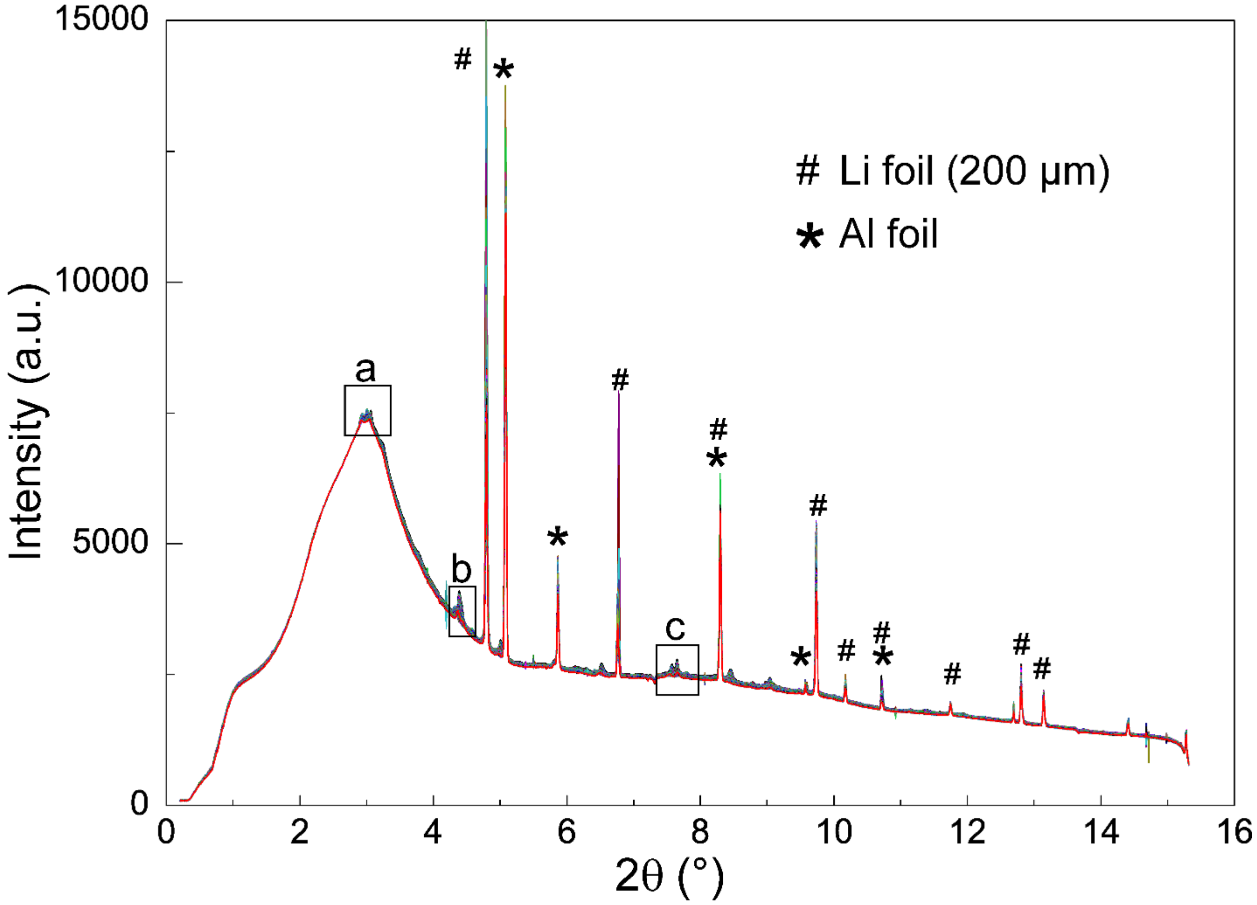

Supplementary Figure 9. All normalized diffraction patterns (~185) obtained for Mn-LCSO during the operando synchrotron XRD experiment. NOTE: Li foil from the counter electrode and Al foil from the cathode sheet dominate the signals of the operando diffraction patterns. Active cathode material with low silicates contributes only weak signal, due to the low area mass loading to ensure good electrochemical performance. Sections (a), (b) and (c) were chosen to further illustrate lattice parameter evolution stemming from active material as shown in Figure 4 of the main text of the paper.

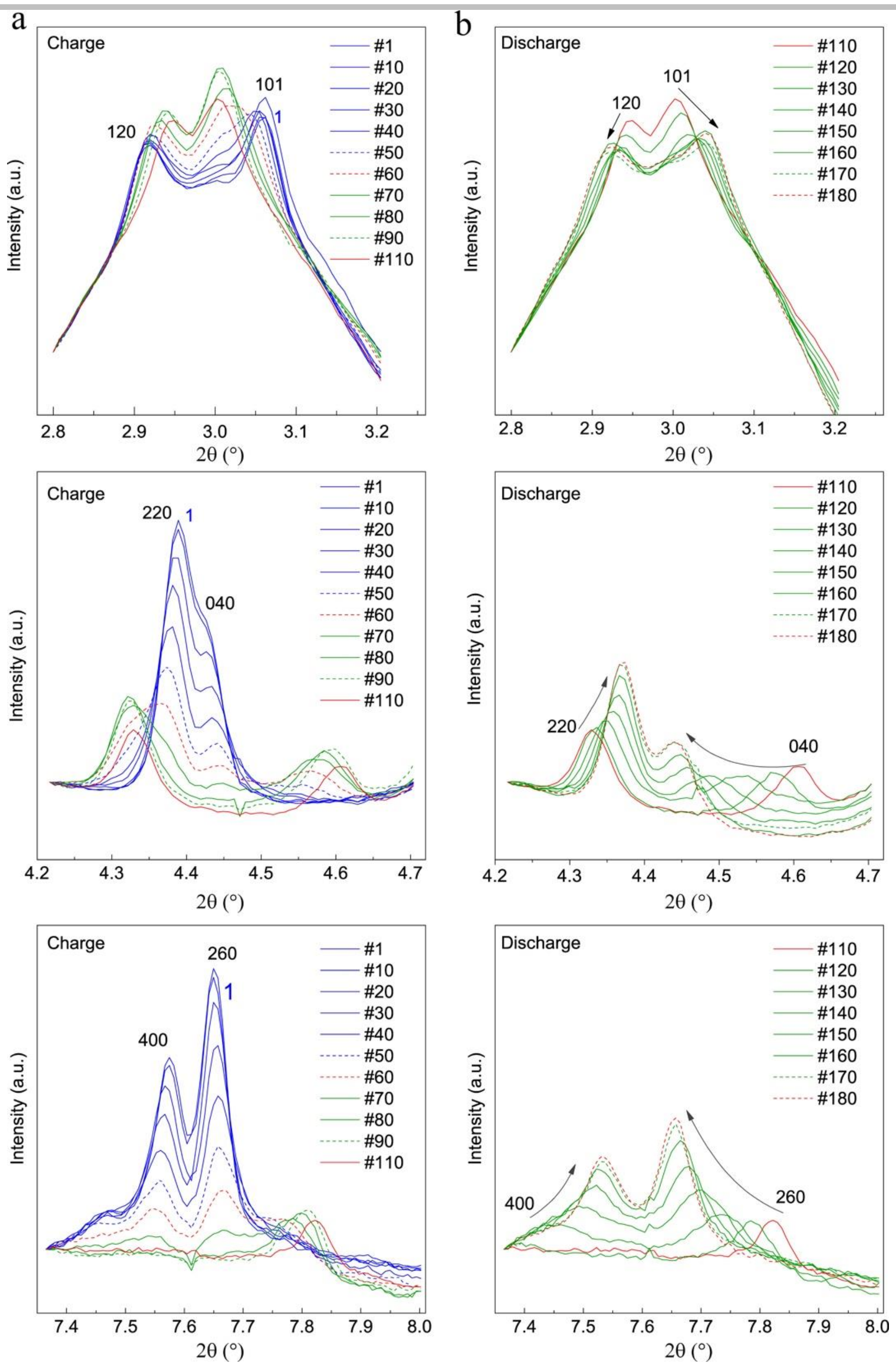

Supplementary Figure 10. Intensity evolution of the operando synchrotron XRD for Mn-LCSO. a. Sections of diffraction patterns during charging. b. Sections of diffraction patterns during discharging. All patterns have been shifted to the same intensity at the lowest scattering angle shown.

Supplementary Table I Refined crystallographic parameters for Li_2CoSiO_4. Although the refinement is rough with Rwp = 10.414%, Rp = 7.414%, GOF=2.95, it indicates the important information of mixed occupation of Li and Co.

Pbn2$_1$(No.33)<br>weight: 69.49%<br>a=6.2894 Å; b=10.7869 Å; c=4.9558 Å					
atom	occupation	Wyckoff symbol	x/a	y/b	z/c
Co/Li/Mn	0.6584/0.2977/0.0439	4a	0.49050	0.16679	0.24300
Si	1	4a	0.25233	0.41757	0.25000
Li/Co/Mn	0.7382/0.2454/0.0164	4a	0.99100	0.15829	0.24100
Li/Co/Mn	0.9641/0.0337/0.0022	4a	0.74162	0.43170	0.16540
O1	1	4a	0.03184	0.34503	0.14780
O2	1	4a	0.24791	0.55787	0.14590
O3	1	4a	0.26002	0.41749	0.58110
O4	1	4a	0.46731	0.34668	0.13960
P2$_1$/n (No. 14)<br>weight: 30.51%<br>a=6.2771 Å; b=10.7977 Å; c=4.9499 Å; β=89.1865					
Co/Mn	0.9375/0.0625	4a	0.50212	0.16255	0.29740
Si	1	4a	0.24700	0.41333	0.31050
Li	1	4a	0.00530	0.15310	0.31720
Li	1	4a	0.22850	0.06600	0.70450
O1	1	4a	0.24750	0.40939	0.63810
O2	1	4a	0.25390	0.55630	0.20710
O3	1	4a	0.03340	0.34090	0.20810
O4	1	4a	0.46040	0.34000	0.20790

Supplementary Note 2
First-principles modeling of structures with O-O dimers
The models are first screened with the HTMad method[9] and then relaxed using VASP[5] by assigning U as 5.0 and J as 0.5 for Co ions, the same as the recent study for Li_2MnO_3.[10] The modeling is based on the ideal Pbn2$_1$ polymorphs without mixed Li/Co occupation, with four formula of Li_2CoSiO_4 per unit cell. Four kinds of cationic site exchange are considered for the totally delithiated phase ($CoSiO_4$). TD1 series, two of the four Co ions occupy two tetrahedral sites opposite to the empty Li sites; TD2 series, two of the four Co ions occupy two empty Li sites; TD3 series, one of the four Co ions occupies the tetrahedral site opposite to the empty Li site; TD4 series, one of the four Co ions occupies one empty Li site. Two of the four Co ions occupying two octahedral sites are considered for the deep delithiated phase of $Li_{1/2}CoSiO_4$. It is worth to note that, the Co ions move away from the octahedral sites to the center of trigonal bipyramids during structural relaxation shown in Fig. 5f. The statistical results are shown in supplementary Table II.

Supplementary Table II First-principles relaxation results. N_{DFT_Cal}, the number of models performed with structural relaxation. $N_{1\ (O\text{-}O)}$, the number of models with one O-O dimer. $N_{1(Si\text{-}O\text{-}O\text{-}Si)}$, the number of models with one O-O dimer, which bridges two Si ions. $N_{m\ (O\text{-}O)}$, the number of models with more than one O-O dimer.

Model	N_{DFT_Cal}	$N_{1\ (O\text{-}O)}$	$N_{1(Si\text{-}O\text{-}O\text{-}Si)}$	$N_{m\ (O\text{-}O)}$
TD1	99	21	11	7
TD2	60	31	17	6
TD3	15	2	2	1
TD4	20	3	2	0
$Li_{1/2}CoSiO_4$	79	22	17	2

Supplementary Note 3
First-principles modeling of XANES
The package XSpectra[7a-c] implemented within Quantum Espresso[11] was used to simulate XANES based on pseudopotential with one 1s electron hole for the excited oxygen ion. The calculations are performed with a $2 \times 1 \times 2$ supercell containing 16 f. u. of $Li_{2-x}CoSiO_4$ (x=0,3/2,2) to avoid spurious interactions of the excited atom with its periodically repeated images. The energy position of the peak contributed by lattice oxygen ion is aligned to the experimental LO peak in Fig. 2.

Supplementary References

[1] aZ. Zhang, Z. Chen, X. Zhang, D. Wu, J. Li, *Electrochim Acta* **2018**, *264*, 166-172; bH. Du, X. Zhang, Z. Chen, D. Wu, Z. Zhang, J. Li, *RSC Advances* **2018**, *8*, 22813-22822; cW. D. Liyuan Huai, Zhifeng Zhang,Xianhui Zhang,Zhiyong Zhang, Zhenlian Chen,Jinlei Wu, Deyu Wang and Jun Li *Electrochim Acta* **2020**, *353*, 136564.

[2] B. H. Toby, R. B. Von Dreele, *J Appl Cryst* **2013**, *46*, 544-549.

[3] G. Ren, N. Zhang, X. Feng, H. Zhang, P. Yu, S. Zheng, D. Zhou, Z. Tian, X. Liu, *Chin Phys B* **2020**, *29*, 016101.
[4] B. Ravel, M. Newville, *J Synchrotron Radiat* **2005**, *12*, 537-541.
[5] aG. Kresse, J. Hafner, *Phys Rev B* **1994**, *49*, 14251-14269; bG. Kresse, J. Furthmu¨ller, *Phys Rev B* **1996**, *54*, 11169-11186.
[6] G. Kresse, D. Joubert, *Phys Rev B* **1999**, *59*, 1758-1775.
[7] aM. Taillefumier, D. Cabaret, A.-M. Flank, F. Mauri, *Phys Rev B* **2002**, *66*, 195107; bC. Gougoussis, M. Calandra, A. Seitsonen, C. Brouder, A. Shukla, F. Mauri, *Phys Rev B* **2009**, *79*, 045118; cC. Gougoussis, M. Calandra, A. P. Seitsonen, F. Mauri, *Phys Rev B* **2009**, *80*, 075102; dO. Bunău, M. Calandra, *Phys Rev B* **2013**, *87*, 205105.
[8] J. P. Perdew, K. Burke, M. Ernzerhof, *Phys Rev Lett* **1996**, *77*, 3865-3868.
[9] Z. Chen, J. Li, *J Comput Chem* **2016**, *37*, 1476-1483.
[10] Z. Chen, J. Li, X. C. Zeng, *J Am Chem Soc* **2019**, *141*, 10751-10759.
[11] aP. Giannozzi, O. Andreussi, T. Brumme, O. Bunau, M. Buongiorno Nardelli, M. Calandra, R. Car, C. Cavazzoni, D. Ceresoli, M. Cococcioni, N. Colonna, I. Carnimeo, A. Dal Corso, S. de Gironcoli, P. Delugas, R. A. DiStasio, A. Ferretti, A. Floris, G. Fratesi, G. Fugallo, R. Gebauer, U. Gerstmann, F. Giustino, T. Gorni, J. Jia, M. Kawamura, H. Y. Ko, A. Kokalj, E. Kucukbenli, M. Lazzeri, M. Marsili, N. Marzari, F. Mauri, N. L. Nguyen, H. V. Nguyen, A. Otero-de-la-Roza, L. Paulatto, S. Ponce, D. Rocca, R. Sabatini, B. Santra, M. Schlipf, A. P. Seitsonen, A. Smogunov, I. Timrov, T. Thonhauser, P. Umari, N. Vast, X. Wu, S. Baroni, *J Phys Condens Matter* **2017**, *29*, 465901; bP. Giannozzi, S. Baroni, N. Bonini, M. Calandra, R. Car, C. Cavazzoni, D. Ceresoli, G. L. Chiarotti, M. Cococcioni, I. Dabo, A. Dal Corso, S. de Gironcoli, S. Fabris, G. Fratesi, R. Gebauer, U. Gerstmann, C. Gougoussis, A. Kokalj, M. Lazzeri, L. Martin-Samos, N. Marzari, F. Mauri, R. Mazzarello, S. Paolini, A. Pasquarello, L. Paulatto, C. Sbraccia, S. Scandolo, G. Sclauzero, A. P. Seitsonen, A. Smogunov, P. Umari, R. M. Wentzcovitch, *J Phys Condens Matter* **2009**, *21*, 395502.
